# Zmym4 is required for early cranial gene expression and craniofacial cartilage formation

**DOI:** 10.3389/fcell.2023.1274788

**Published:** 2023-10-03

**Authors:** Karyn Jourdeuil, Karen M. Neilson, Helene Cousin, Andre L. P. Tavares, Himani D. Majumdar, Dominique Alfandari, Sally A. Moody

**Affiliations:** ^1^ Department of Anatomy and Cell Biology, George Washington University, School of Medicine and Health Sciences, Washington, DC, United States; ^2^ Department of Veterinary and Animal Sciences, University of Massachusetts, Amherst, MA, United States

**Keywords:** neural border, neural plate, preplacodal ectoderm, neural crest, otic vesicle, *xenopus*, Six1.

## Abstract

**Introduction:** The Six1 transcription factor plays important roles in the development of cranial sensory organs, and point mutations underlie craniofacial birth defects. Because Six1’s transcriptional activity can be modulated by interacting proteins, we previously screened for candidate interactors and identified zinc-finger MYM-containing protein 4 (Zmym4) by its inclusion of a few domains with a *bona fide* cofactor, Sine oculis binding protein (Sobp). Although Zmym4 has been implicated in regulating early brain development and certain cancers, its role in craniofacial development has not previously been described.

**Methods:** We used co-immunoprecipitation and luciferase-reporter assays in cultured cells to test interactions between Zmym4 and Six1. We used knock-down and overexpression of Zmym4 in embryos to test for its effects on early ectodermal gene expression, neural crest migration and craniofacial cartilage formation.

**Results:** We found no evidence that Zmym4 physically or transcriptionally interacts with Six1 in cultured cells. Nonetheless, knockdown of endogenous Zmym4 in embryos resulted in altered early cranial gene expression, including those expressed in the neural border, neural plate, neural crest and preplacodal ectoderm. Experimentally increasing Zmym4 levels had minor effects on neural border or neural plate genes, but altered the expression of neural crest and preplacodal genes. At larval stages, genes expressed in the otic vesicle and branchial arches showed reduced expression in Zmym4 morphants. Although we did not detect defects in neural crest migration into the branchial arches, loss of Zmym4 resulted in aberrant morphology of several craniofacial cartilages.

**Discussion:** Although Zmym4 does not appear to function as a Six1 transcriptional cofactor, it plays an important role in regulating the expression of embryonic cranial genes in tissues critical for normal craniofacial development.

## 1 Introduction

Six1 is a key transcriptional regulator of many developmental processes and several single nucleotide mutations are causative for Branchio-oto-renal syndrome (BOR) and Deafness, autosomal dominant 23 (DFNA23) (reviewed in (Moody and Saint-Jeannet, 2014; Moody and LaMantia, 2015; Riddiford and Schlosser, 2016; Smith, 2018; Streit, 2018; Schlosser, 2021)). Six1 can function as either a transcriptional activator or repressor depending upon the binding of different cofactors to its protein interaction domain (Silver et al., 2003; Brugmann et al., 2004; Anderson et al., 2012). Previously, we identified zinc-finger MYM-containing 4 (Zmym4) as a candidate Six1 cofactor (Neilson et al., 2010), based on structural similarities to Box2 and Box3 of Sine oculis-binding protein (Sobp), a cofactor discovered in *Drosophila* that also is expressed in frogs and mice (Kenyon et al., 2005; Tavares et al., 2021). While a closely related MYM-containing protein (Zmym2) interacts with Six4 during renal development (Connaughton et al., 2020), nothing is known about possible interactions between Zmym4 and Six transcription factors, including Six1, or whether Zmym4 is involved in craniofacial development.

The function of ZMYM4 has only recently begun to be investigated in human health. Mutations in ZMYM4 are associated with severe childhood obesity (Greenhill, 2020; Marenne et al., 2020; Regan and Shah, 2020), schizophrenia (Xiang et al., 2020), heritable substance use disorders (Zhang et al., 2022), heritable sleep disorders (Spada et al., 2016), and cancers (Goyal et al., 2017; Goyal et al., 2017; Goyal et al., 2019; Goyal et al., 2021; Moon et al., 2021; Yu et al., 2023). Despite keen interest in the newly identified role of ZMYM4 in human disease, little is known whether it has a role in early developmental processes, despite its abundant expression, particularly in craniofacial anlagen, during embryogenesis (Neilson et al., 2010).

During *Xenopus* development*, Zmym4* is expressed in the neural plate and neural tube, preplacodal ectoderm (PPE) and cranial sensory placodes, neural crest (NC), branchial arches (BAs), somites and nephric mesoderm (Neilson et al., 2010). Similar expression patterns have been described at E10.5 in the mouse (Gray et al., 2004) and in the E15.5 mouse genitourinary tract (GUDMAP; www.gudmap.org, 2004). These tissues also express *Six1* during development (reviewed in (Moody and Saint-Jeannet, 2014; Moody and LaMantia, 2015; Riddiford and Schlosser, 2016; Streit, 2018)), suggesting that Zmym4 and Six1 may have the opportunity to interact during critical periods of craniofacial development. Since Zmym4 contains two domains with homology to *Drosophila* Sobp (Box2 and Box3; (Kenyon et al., 2005)) and *Xenopus* Sobp interacts with Six1 and is required for craniofacial development (Tavares et al., 2021), we assessed whether Zmym4 also interacts with Six1 and/or modifies its transcriptional activity.

Herein, we show by assays in HEK293T cells that Zmym4 does not co-immunoprecipitate or transcriptionally interact with Six1. Nonetheless, knockdown of endogenous Zmym4 in embryos resulted in altered early cranial gene expression, including genes expressed in the neural border (NB), neural plate (NP), NC and PPE. Experimentally increasing Zmym4 levels had minor effects on NB or NP genes, but altered the expression of NC and PPE genes. At larval stages, genes expressed in the otic vesicle and BAs showed reduced expression in Zmym4 morphants. Although we did not detect defects in the migration of NC cells into the BAs, loss of Zmym4 resulted in aberrant morphology of several craniofacial cartilages. Thus, although Zmym4 does not appear to function as a Six1 transcriptional cofactor, herein we show that it plays an important role in regulating the expression of embryonic cranial genes in tissues critical for normal craniofacial development.

## 2 Materials and methods

### 2.1 Plasmid constructs

Two partial *Xenopus laevis zmym4* (BC068960, BC108780) plasmids were purchased from Dharmacon. To generate a full-length *zmym4* plasmid construct, the BC108780 ORF was subcloned into the BamHI (Thermo Fisher Scientific Cat# FD0054) and XbaI (New England Biolabs Cat# R0145S) sites of pCS2+ (*pCS2+zmym4*) using the Clone EZ PCR cloning kit (GenScript Gene Synthesis Cat# L00339). PCR was then used to amplify the region of the BC068960 plasmid spanning from the internal SpeI restriction site to the 3′ BamHI, removing the 3′ UTR present in the BC068960 plasmid. The PCR-generated second half of the *zmym4* ORF was then ligated into the SpeI (New England Biolabs Cat# R0133S) and 3′ BamHI sites of the *pCS2+zmym4* plasmid (*pCS2+zmym4. L*). To generate both morpholino (MO)-sensitive and MO-insensitive HA-tagged full-length *zmym4* constructs, gBlock fragments were designed (Supplementary Figure S1A) and purchased from Integrative DNA Technologies. These contained the 5′ restriction site, a restriction site found within the ORF, and either the 5′UTR region containing the binding site for the translation blocking MO or an edited version of the 5′UTR removing the binding site for MO1 and editing the nucleotide sequence for MO2 to prevent MO binding but conserving the amino acid sequence. After confirming that these 5′ gBlock fragments were successfully ligated into *pCS2+zmym4. L* (*pCS2+zmym4. L-5′UTR*; *pCS2+zmym4. L-5′MOinsensitive*) and confirmed by full-length sequencing, the process was repeated at the 3′ end to insert a gBlock fragment containing a 5′ restriction site found within the ORF, the 3′ restriction site, and an HA-tag just before the TAA stop codon (*pCS2+zmym4. L-3′HA*; *pCS2+zmym4. L-5′UTR3′HA*; *pCS2+zmym4. L-5′MOinsensitive3′HA*). The inserted 3′HA sequence was confirmed in each construct by full-length sequencing and expression validated by Western blot (Supplementary Figure S1B).

### 2.2 Cell culture

HEK293T/17 cells (American Type Culture Collection (ATCC) CRL-11268, RRID:CVCL_0063) were cultured in Dulbecco’s modified Eagle medium (DMEM; Cytiva Life Sciences Cat# SH30022.01) supplemented with 10% fetal bovine serum (FBS; Gibco; Thermo Fisher Scientific Cat# 10437-028) and penicillin-streptomycin (PenStrep; Gibco; Thermo Fisher Scientific Cat# 15070-063). Cells were plated into 24-well plates for luciferase assays and into 6-well plates for co-immunoprecipitation (Co-IP) experiments. Cells were transfected according to the manufacturer’s protocol using Lipofectamine 3,000 (Invitrogen; Thermo Fisher Scientific Cat# L3000-015). Cells were processed for each assay 48 h after transfection.

### 2.3 Co-immunoprecipitation

HEK293T cells were transfected with 1.5 μg of *pCS2+3′MycSix1, pCS2+zmym4. L-3′HA*, or *pCS2+3′MycSix1* and *pCS2+zmym4. L-3′HA.* Cytoplasmic proteins were extracted from cells 48 h after transfection using a volume of NE-PER Nuclear and Cytoplasmic Extraction Buffer CER I (Thermo Fisher Scientific Cat# 78833) according to the size of the cell pellet as per manufacturer instructions with added Halt Protease Inhibitor Cocktail with EDTA (Thermo Fisher Scientific Cat# 78430). After collecting the cytoplasmic fraction, the remaining nuclear cell pellet was lysed using NER buffer with added Halt and EDTA, according to the manufacturer protocol. The concentration of the cytoplasmic and nuclear supernatants was determined using the Pierce BCA Assay Kit (Thermo Fisher Scientific Cat# 23225). The same concentration of each sample was then subjected to immunoprecipitation using the Pierce anti-c-Myc magnetic beads (Thermo Fisher Scientific Cat# 88842) or the Pierce anti-HA magnetic beads (Thermo Fisher Scientific Cat# 88836). Proteins were washed according to the manufacturer protocol for each of the beads and eluted using the Pierce Lane Marker Non-Reducing Sample Buffer (Thermo Fisher Scientific Cat# 39001) for the anti-C-Myc beads and a 2 μg per ml concentration of HA peptide (Thermo Fisher Scientific Cat# 26184) following the gentle elution protocol in the manufacturer protocol for the anti-HA beads. The eluted proteins were reduced using 100 mM dithiothreitol (DTT; Thermo Fisher Scientific Cat# R0861) (anti-C-Myc beads) or Laemmli Sample Buffer (Bio-Rad Laboratories Cat# 161-0747 with 2% β-mercaptoethanol (BME; Bio-Rad Laboratories Cat# 161-0710) (anti-HA beads) at 100 °C for 10 min prior to SDS-PAGE and Western blot using 8%–16% mini-protean TGX gels (Bio-Rad Laboratories Cat# 456-1104). For control experiments, 30 μL of each lysate in CER or NER buffer was diluted in Laemmli Sample Buffer with 2% BME, incubated at 100 °C for 10 min prior to SDS-PAGE and Western blot. Immobilon-FL PVDF membranes (Fisher Scientific Cat# IPFL00010) were probed with mouse anti-Myc (9B11 at 1:1,000; Cell Signaling Technology Cat# 2040, RRID:AB_2148465), rabbit anti-HA (C29F4 at 1:1,000; Cell Signaling Technology Cat# 3724, RRID:AB_1549585), rabbit anti-Zmym4 (1:1,000; Lifespan Biosciences (LSBio) Cat# LS-C193170/200,169), rabbit anti-Six1 (D5S2S at 1:1,000; Cell Signaling Technology Cat# 16960, RRID:AB_2798773), or mouse anti-HA (6E2 at 1:1,000; Cell Signaling Technology Cat# 2999, RRID:AB_1264166). For all blots, mouse anti-αTubulin (DM1A at 1:5,000; Novus Biologicals Cat#NB110-93473, RRID:AB_1236645) was used as a control and the IRDye 680RD donkey anti-rabbit IgG (1:5,000; LI-COR Biosciences Cat# 925-68073, RRID:AB_2716687) and IRDye 800 CW goat anti-mouse IgG (1:5,000; LI-COR Biosciences Cat#925-32210, RRID:AB_2687825) were used as secondary antibodies. The Zmym4 antibody was validated by Western blot to generate a protein of the expected size and to overlap with the HA-antibody positive band generated by the *pCS2+zmym4-3′HA* construct. Experiments were repeated at least three independent times. Blots were scanned using the LI-COR Odyssey Classic Infrared Imaging System and analyzed using LI-COR Image Studio Software (RRID:SCR_015795).

### 2.4 Luciferase assay

HEK293T cells were transfected with 200 ng of *pGL3-6xMEF3-Firefly* luciferase, a Six1-inducible reporter containing 6 Six1 binding sites (Ford et al., 2000), and 100 ng of *pRL-TK-Renilla* luciferase reporter in addition to different combinations of *pCS2+* (empty vector control), *pCS2+3′FlagSix1*, *pCS2+5′MycEya1,* and *pCS2+zmym4. L* (400 ng each). Plasmids encoding Six1 BOR variants are as previously reported (Shah et al., 2020). At 48 h post-transfection, cells were resuspended directly in 100 μL of Passive Lysis Buffer (from the Dual Luciferase Assay Kit; Promega Corporation Cat# E1980) for 1 h at room temperature and then frozen at −80 °C overnight. The following day, 20 μL of lysate was analyzed using the Dual Luciferase Assay kit following the manufacturer protocol. Experiments were repeated at least 5 times. ANOVA with Tukey *post hoc* multiple comparisons test was performed using GraphPad Prism 9 software (RRID:SCR_015795). Expression of exogenous proteins from the transfected plasmids was confirmed by standard Western blotting using the antibodies described for Co-IP with the exception of Eya1, which was identified using the mouse anti-Myc and anti-Eya1 antibodies (1:1,000; Proteintech Cat# 22658-1-AP, RRID:AB_2879145) and Six1, which was identified using the mouse anti-DYKDDDDK (9A3 at 1:1,000; Cell Signaling Technology Cat# 8146, RRID:AB_10950495) or rabbit anti-Six1 antibodies described above.

### 2.5 *In vitro* synthesis of RNAs

mRNAs encoding *X. laevis zmym4* (*pCS2+zmym4. L* or *pCS2+zmym4. L-5′MOins3′HA*) and a nuclear-localized *β*-*galactosidase* (*nβgal*) lineage tracer were synthesized *in vitro* according to the manufacturer protocol (SP6 mMessage mMachine kit; Thermo Fisher Scientific Cat# AM1340). Antisense RNA DIG-labeled probes for *in situ* hybridization (ISH) were synthesized *in vitro* (MEGAscript SP6 Transcription Kit; Invitrogen, Thermo Fisher Scientific Cat# AM1330, as previously described (Yan et al., 2009).

### 2.6 Embryo microinjections

Fertilized *X. laevis* embryos were obtained as previously described and chosen at the 2-cell stage to accurately identify the dorsal and ventral animal blastomeres (Klein, 1987; Miyata et al., 1987; Moody, 2018). When selected embryos reached the 8-cell stage, the dorsal-animal and ventral-animal blastomeres that predominantly give rise to the neural crest and cranial placodes (Moody and Kline, 1990) were each microinjected with 1 nL of mRNA (100 pg mixed with 100 pg *nβgal*) or MOs (4.5 ng of an equimolar mixture of MO1+MO2) according to standard methods (Moody, 2018). Only one side of the embryo was microinjected rendering the uninjected side as an internal control. Embryos were cultured in diluted Steinburg’s solution at 16 °C until fixation.

### 2.7 Morpholino oligonucleotides

To knockdown endogenous levels of Zmym4 protein *in vivo*, two lissamine-tagged translation-blocking antisense morpholino oligonucleotides (MOs) were purchased (GeneTools, LLC) with the following sequences: Zmym4 MO1: 5′-GAC​TGT​GAT​TAG​TAT​CAG​CAT​CCA​T-3′ and Zmym4 MO2: 5′-TAT​TCC​ATC​GCC​CGG​TCA​GCC​CGG​T-3’. These MOs bind in the 5′UTR directly adjacent to the starting ATG or to the beginning of the coding region of both the L- and S-homoeolog of *X. laevis zmym4* (Supplementary Figure S1A). To verify the ability of the MOs to block *zmym4* translation, both cells of a 2-cell *X. laevis* embryo were injected with an equimolar mixture of the Zmym4 MOs. At stage 26 (Nieuwkoop and Faber, 1994), MO-injected and sibling control embryos were snap frozen on dry ice and stored overnight at −80 °C. They were lysed in 500 μL of RIPA buffer with added HALT and EDTA protease inhibitors and mechanically disrupted using a 1.5 mL pestle. To remove the yolk proteins, samples were sequentially centrifuged at 500 *g*, 750 x *g*, 1000 x *g*, and 1250 x *g* at 4 °C and the supernatant transferred to a clean 1.5 mL microcentrifuge tube between each centrifugation step. Expression of Zmym4 protein was determined Western blot, as described above (Supplementary Figure S1C) and protein levels normalized to αTubulin to determine knockdown efficacy (Supplementary Figure S1D). The specificity of the knockdown was determined by injecting embryos at the 8-cell stage with an equimolar mixture of Zmym4 MOs, as above, with or without mRNA encoding *zmym4-5′MOins3′HA,* which is insensitive to MO binding (Supplementary Figure S1A). These embryos were processed for *in situ* hybridization (see below) and assessed for the percentage of embryos showing reduced *sox9* expression (Supplementary Figure S1E).

### 2.8 Histochemistry and *in situ* hybridization

Embryos were cultured to early neural plate (stage 13), neural fold (stages 16–18), or larval (stages 28–32) stages (Nieuwkoop and Faber, 1994), fixed in 4% paraformaldehyde (PFA; MP Biomedicals Cat# 150146), stained for *βgal* histochemistry (if mRNA-injected), and processed for *in situ* hybridization (ISH) as previously described (Yan et al., 2009). Only embryos in which the fluorescent MOs or nβGal lineage tracer were located in the appropriate tissue domains were analyzed for the expression of *dlx5*, *foxd3*, *irx1*, *msx1*, *pax3*, *six1*, *sox2*, *sox9*, *sox11*, *tbx1,* or *tfap2α*. For each gene, the staining intensity and size of the expression domain were compared between the injected, lineage-labeled side to the control, uninjected side of the same embryo, thus minimizing inter-embryo variation that might occur with differences in developmental stages or experimental batch. Embryos that showed a more intense staining pattern or larger expression domain on the injected side compared to control side were scored as “increased.” Embryos that showed a less intense staining pattern or smaller expression domain on the injected side compared to the control side were scored as “decreased.” The percentages of embryos that showed “increased” expression, “decreased” expression, or “no change” between injected and control sides of the same embryo were calculated for each gene, and differences in the frequency assessed for significance (*p* < 0.05) by the Chi-square test using GraphPad Prism 9. Embryos were collected from at least three different sets of outbred, wild-type parents to account for genetic diversity for each of the genes assessed in both MO and mRNA treatment conditions.

### 2.9 Neural crest migration assays

The extent of cranial NC migration was assessed using two approaches: *in vivo* and *in vitro*. *In vivo* studies were performed as previously described (Cousin et al., 2012). Briefly, the dorsal animal blastomere of the 8-cell embryo, which is the major precursor of the NC (Moody and Kline, 1990), was microinjected with 100 pg of membrane-associated GFP (*mbGFP*) mRNA alone or in combination with either 1.3 ng of Zmym4 MOs equimolar mixture (as above) or 1 ng of Sobp MOs (characterized in (Tavares et al., 2021)). When embryos reached neural crest migratory stages (stages 22-23), those expressing the GFP lineage marker in the dorsal anterior region were scored for the presence or absence of fluorescently labeled NC cells in the NC migration pathways into the BAs; partial migration was scored as presence. For each experiment, the results from embryos in which only lineage tracer was injected (control) were used as a normalizer and set to 100%. The control and morphant numbers were compared using a two-tailed, paired Student’s t-test. *In vitro* studies were performed as described in Cousin and Alfandari (2018). One cell of the embryo was injected at the 2-cell stage with either 200 pg of *mbGFP* mRNA alone or in combination with either 2.5 ng of Zmym4 MOs equimolar mixture (as above) or 2 ng of Sobp MOs. At stage 15, explants of fluorescently labeled cranial NC were dissected from the embryo, individually placed into a 96-well plate coated with 20 μg/mL of Fibronectin (FN), and cultured at 18 °C in Danilchik medium containing 50 μg/mL gentamicin. Once the explants attached to the plate (at ∼1 h post explant), time lapse images were taken using a Keyence microscope at a rate of one image every 3 min for ∼10–11 h. Movies were assembled using Keyence BZ-X Analyser software. The surface area occupied by the explant at the beginning and at the end of the movie were determined manually using Fiji software. The ratio of these areas (end/beginning) were calculated and normalized to the ratio obtained from control explants and compared using a two-tailed, paired Student’s t-test.

### 2.10 TUNEL staining

To determine if changes in NC gene expression might result from apoptosis, embryos were injected on one side with Zmym4 MOs as above and cultured to larval stages (stage 35–36). Embryos were fixed and processed for TUNEL staining as wholemounts according to the manufacturer protocol (ApopTag *In Situ* Apoptosis Detection Kit; EMD Millipore Cat# S7101) as adapted by (Zaghloul and Moody, 2007). After staining, the skin was removed with fine forceps and the number of TUNEL-positive cells in all four BAs counted on the control and MO-injected sides of each embryo. The numbers were compared using a two-tailed, paired *t*-test (*p* < 0.05) using GraphPad Prism 9.

### 2.11 Cartilage staining

Embryos were injected with Zmym4 MOs as above and grown to tadpole stages (stages 45–47) when the cranial cartilages have formed. Tadpoles were fixed in 4% PFA, dehydrated to 100% ethanol, stained overnight in Alcian blue (Sigma-Aldrich Cat# A5268) and cleared in a KOH (Emsure; EMD Millipore Cat# 1310-S8-3):glycerol (Fisher Scientific Cat# BP229-1) series, as previously described (Walker and Kimmel, 2007; Tavares et al., 2021). Morphological dysmorphologies in cartilages contributing to the lower jaw (infrarostral, Meckel’s, ceratohyal), otic capsule, and pharyngeal apparatus (ceratobranchial cartilages) (Svensson and Haas, 2005) were each visually scored as “normal”, “mildly defective”, or “severely defective” as previously described (Keer et al., 2022). Briefly, a defect was scored as “mild” if slightly displaced or reduced in size and “severe” if the cartilage was significantly displaced or significantly reduced or missing. The frequencies of each phenotype per cartilaginous element were calculated across three independent experiments using the Chi-squared test (*p* < 0.05). To quantify the extent of the disruption of the craniofacial cartilages, images of 76 Zmym4 morphants were taken at 32X using the Olympus cellSens software (RRID:SCR_014551) on an Olympus SZX16 stereomicroscope. These images were imported into Adobe Photoshop 2022 (RRID:SCR_014199) and the scale bar used to set the measurement scale for quantification (174 pixels = 200 μm). The length of both left and right Meckel’s, ceratohyal, and otic capsule cartilages, and the length of the unpaired infrarostral cartilage were measured as illustrated (Supplementary Figure S2). These measurements were then exported into GraphPad Prism 9 and the measurements for each element analyzed for normal distribution with the Shapiro-Wilks test. As the measurements for one of the elements were not normally distributed, each data set was analyzed for significant differences between the morphants and sibling controls using a Mann-Whitney test (*p* < 0.05).

## 3 Results

**FIGURE 1:**
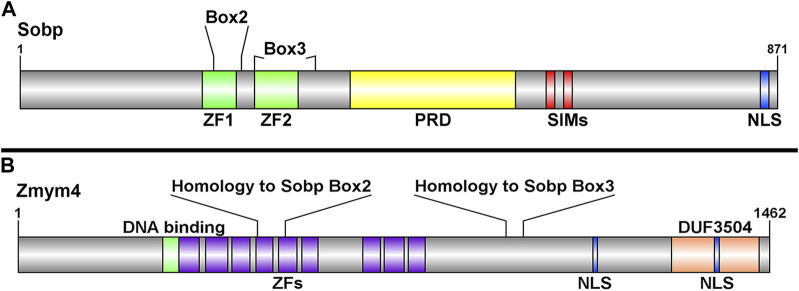
Schematic representations of the Sobp and Zmym4 proteins highlighting their different domains. **(A)** Sobp protein structure adapted from Tavares et al., 2021 highlighting Box2 and Box3 identified by Kenyon et al., 2005. Sobp contains two zinc fingers (ZF1, ZF2), a proline rich domain (PRD), two SUMO-interacting motifs (SIM), and a functional nuclear localization signal (NLS). **(B)** The Zmym4 protein contains nine zinc finger MYM-containing domains (purple boxes) identified in human ZMYM4 and aligned by sequence homology (Supplementary Figure S3). The areas of sequence homology with Sobp Box2 and Box3 are indicated. Zmym4 contains two putative NLSs, a glucocorticoid-like DNA binding domain (green box), and a C-terminal DUF3504 domain. The NLSs were identified using cNLS mapper (Kojima and Jurka, 2011), the DNA-binding and DUF3504 domains were identified using InterPro (https://www.ebi.ac.uk/interpro/) and the protein structures were generated using DOG2.0.1 (http://dog.biocuckoo.org/). Numbers on the right of each schematic indicate amino acid length of the protein.

To identify novel Six1-interacting proteins that might be causative candidates for BOR, we previously screened the fly interactome for Sine oculis interacting proteins whose orthologs are also expressed in *Xenopus* embryos (Neilson et al., 2010). Zinc finger MYM-binding protein 4 (Zmym4) was identified as a candidate by its sequence similarity to a few domains found in *Drosophila* Sobp (Box2 and Box3) (Kenyon et al., 2005), which were confirmed to be present in *Xenopus* Sobp (Tavares et al., 2021). *Xenopus* Sobp contains two zinc fingers, a proline rich domain, two SUMO-interacting motifs (SIMs), and a functional nuclear localization signal (NLS) (Figure 1A). Box2 and Box3 span the two zinc finger domains and share sequence homology with two regions in Zmym4 (Figure 1B). *Xenopus laevis* Zmym4, which contains nine zinc finger domains, is highly conserved between the long and short homoeologs, the *Xenopus tropicalis* and human homologs, and the *Drosophila* ortholog Without children (Woc; Supplementary Figure S3). Zmym4 has a predicted glucocorticoid-like DNA binding domain that spans part of the first zinc finger domain as well as a DUF3504 domain in the C-terminal region (Figure 1B), which is related to the tyrosine recombinase element of Crypton DNA transposon elements suggesting it may function as a transcriptional activator and/or repressor (Kojima and Jurka, 2011). The DUF3504 domain also exhibits *in silico* structural similarity and likely evolutionary conservation with the BEN family DNA-binding domains (Pan et al., 2023), also suggesting a nuclear function. A putative NLS was detected within the DUF3504 domain (Figure 1B). Furthermore, Zmym4 has been shown to be a B-MYB binding protein and that it is SUMOylated, suggesting that it contains currently unannotated, non-canonical SIMs consistent with what has been described for ZMYM2 and ZMYM3 (Anderson, 2015; Cibis et al., 2020). These sequence similarities to other nuclear factors suggest that Zmym4 may function as a transcription factor.

### 3.1 Zmym4 is unlikely to function as a Six1 co-factor

A number of cofactors have been identified to regulate Six1 activity. Eya1 is required for Six1 to function as transcriptional activator, whereas Groucho (*a.k.a*., Grg4, Tle4) causes Six1 to function as a transcriptional repressor (Silver et al., 2003; Brugmann et al., 2004; Anderson et al., 2012). Other Six1-binding proteins (*i.e.*, Pa2G4, Mcrs1, Sobp) act to reduce the activity of the Six1-Eya1 transcriptional complex (Neilson et al., 2017; Neilson et al., 2020; Tavares et al., 2021; Keer et al., 2022).

**FIGURE 2:**
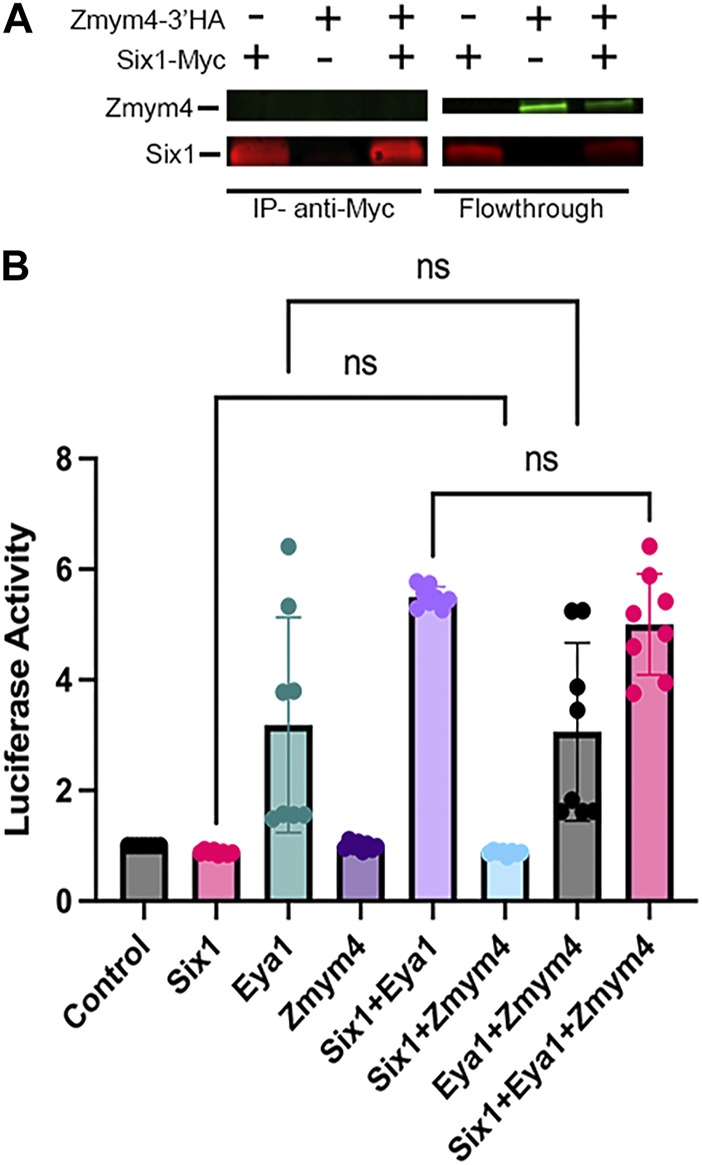
Zmym4 does not bind to Six1 or alter the transcriptional activity of Six1, Eya1, or the Six1-Eya1 transcriptional complex in cultured cells. **(A)** HEK293T cells were transfected with either Myc-tagged Six1, HA-tagged Zmym4, or both and were tested for co-immunoprecipitation (Co-IP) with anti-Myc tagged magnetic beads. In the Co-IP assay (IP—anti-Myc), when Six1 was transfected alone, it bound to the Myc-tagged beads (left lane: Zmym4-3′HA (−), Six1-Myc (+)), whereas when Zmym4 was transfected alone, it did not bind, as expected (middle lane: Zmym4-3′HA (+), Six1-Myc (−)). When both were transfected, (right lane: Zmym4-3′HA (+), Six1-Myc (+)), Six1 was immunoprecipitated by the beads but Zmym4 was not detected in the sample. The flowthrough lanes contained detectable Myc-tagged Six1 but no HA-tagged Zmym4 (left lane), detectable HA-tagged Zmym4 but no Myc-tagged Six1 (middle lane) and detectable Myc-tagged Six1 and HA-tagged Zmym4 (right lane). **(B)** Luciferase activity of the pGL3-6XMEF-luciferase reporter in HEK293T cells transfected with different combinations of empty vector control, Six1, Eya1, and Zmym4 plasmids. Data were normalized to Renilla expressed by a constitutively active promoter. There was no significant difference between the transcriptional activity of Six1 alone, Zmym4 alone and Six1+Zmym4, between Eya1 alone and Eya1+Zmym4, or between Six1+Eya1 and Six1+Eya1+Zmym4 (ns = *p* > 0.05).

Neilson et al. (2010) showed that *Zmym4* is expressed in similar embryonic tissues as both *Six1* and *Eya1*, including the branchial arches (BAs), neural crest (NC) and cranial placodes. This co-expression, along with its sequence similarity to Sobp (Figure 1), suggest that Zmym4 may interact with Six1 during early cranial development. To determine whether Zmym4 can interact with Six1, HEK293T cells were transfected with either Myc-tagged Six1, HA-tagged Zmym4, or both and the lysates assayed by Co-IP using either anti-c-Myc tagged beads or anti-HA tagged beads. Despite performing multiple replicates under different conditions (*i.e.*, performing the IP for either Myc- or HA-tag, different concentrations of input protein, and different elution conditions), we were unable to detect binding of Zmym4 to Six1 in this assay (Figure 2A). It is notable that the flowthrough from the Co-IP double transfection shows a lower level of Zmym4 compared to the flowthrough from the single Zmym4-3′HA lane. This may indicate that the co-expression of Six1 in the doubly transfected cells affects Zmym4 translation or stability. However, in previous reports we have seen this same reduction even when there is clear evidence for a Six1-cofactor interaction (Neilson et al., 2017; Neilson et al., 2020; Tavares et al., 2021). Therefore, we do not believe that the lack of Zmym4 binding to Six1 in the Co-IP lane is due to a reduction in Zmym4 levels. However, this requires further biochemical validation.

Despite the lack of direct binding between Zmym4 and Six1, a recent publication identified that single nucleotide polymorphism (SNP) variants of Zmym4 are able to act on nearby genes (Spada et al., 2016); therefore, it was important to determine whether Zmym4 may have an indirect role in regulating the transcriptional activity of Six1, Eya1, or the Six1-Eya1 transcriptional complex. To assess whether Zmym4 could modulate Six1 transcriptional activity, HEK293T cells were co-transfected with a Six1-inducible reporter (Ford et al., 2000) and different combinations of Six1, Eya1, and/or Zmym4 encoding plasmids. As demonstrated previously (Patrick et al., 2009; Tavares et al., 2021), in the presence of Eya1, Six1 induced a significant increase in luciferase activity over control plasmid or Six1 alone (Figure 2B). In contrast, neither Zmym4 alone or in the presence of Six1 caused a significant change in luciferase activity over that of the control or Six1 alone (Figure 2B), indicating that Zmym4 does not alter Six1 transcriptional activity in this assay. Next, as the HEK293T cells have a low level of endogenous Six1 expression, reflected in the moderate activation of the Six1-inducible luciferase reporter with the transfection of only Eya1 (Tavares et al., 2021) (Figure 2B), we investigated whether Zmym4 was able to alter the transcriptional activity of Eya1. We again found that there was no significant difference in the transcriptional activity of the Six1-inducible luciferase reporter activity when assayed with Eya1 alone or in the presence of Zmym4 (Figure 2B). Finally, we assayed whether Zmym4 could alter the activity of the Six1-Eya1 transcriptional complex. We noted no significant difference in luciferase reporter activity in the presence of Six1+Eya1 with or without the addition of Zmym4 (Figure 2B). Consistent with a previous study that showed BOR variants of Six1 are transcriptionally deficient (Tavares et al., 2021), we did not detect any effect on transcriptional activity when Zmym4 was transfected with a Six1 BOR variant either alone or in the presence of Eya1 (Supplementary Figure S4). Together, these data show that despite some sequence similarity to Sobp, Zmym4 is unlikely to function as a Six1 cofactor as it neither binds to Six1 nor alters Six1 or Eya1 transcriptional activity in the *in vitro* assays we performed.

### 3.2 Zmym4 affects cranial neural crest and placode gene expression

Although we did not detect any evidence that Zmym4 could bind to Six1 or modulate its transcriptional activity in a cell line, its expression in the developing NC and cranial placodes suggests a cranial developmental function. Therefore, we assayed whether loss- or gain-of-function of Zmym4 in embryos could affect the expression of genes that regulate early cranial development. First, to determine whether Zmym4 is required for the formation of the cranial NC or PPE, we decreased the endogenous levels of Zmym4 with specific translation blocking MOs injected into their precursor blastomeres at the 8-cell stage (Moody and Kline, 1990). These embryos were then assessed for the expression of genes that are expressed in the NB (*msx1, tfap2α, pax3*), NP (*sox2, sox11, irx1*), cranial NC (*foxd3, sox9*), or PPE (*six1, sox11, irx1, sox9*). The size of the expression domain and/or intensity of the ISH color reaction were then compared between the MO-injected and control side of each embryo and scored as either “increased” (*e.g.,* larger or more intense), “decreased” (*e.g.*, smaller or less intense), or “no change”.

**FIGURE 3:**
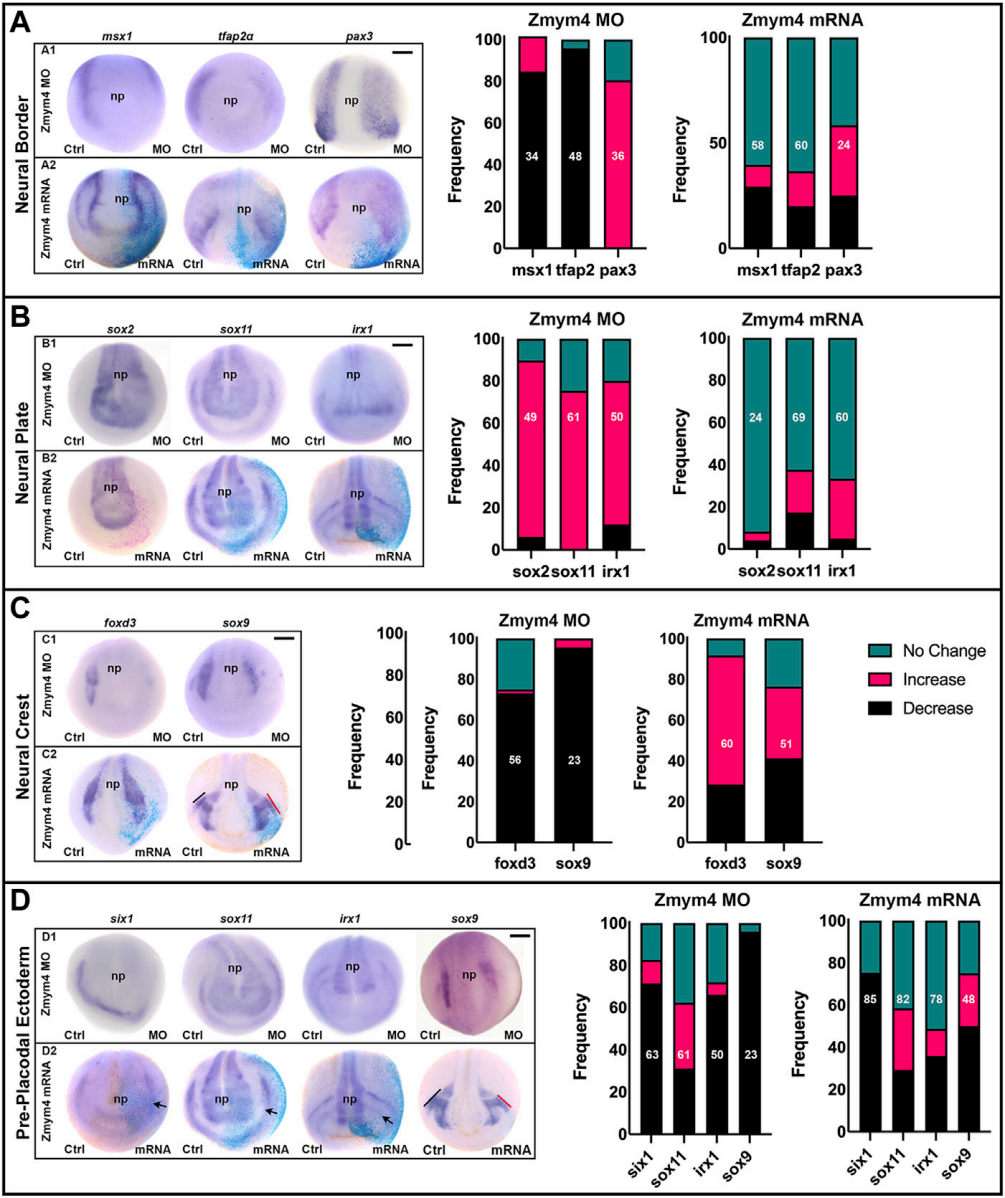
Altering the levels of Zmym4 in embryos differentially altered the expression of early cranial genes. **(A)** In the neural border zone, knockdown of Zmym4 by MO injection (A1) resulted in decreased expression of *msx1* and *tfap2α* and broader expression of *pax3* on the MO-injected side compared to the control (Ctrl) side of the same embryo. Increased expression of Zmym4 by mRNA injection (A2) caused no change in expression in most embryos. The percentages of embryos that showed “no change” (green), “increased” (pink), and “decreased” (black) expression on the injected side compared to the control side of the same embryo are reported in the bar graphs; the number of embryos examined for each gene is indicated within the bar. **(B)** In the neural plate, knockdown of Zmym4 (B1) resulted primarily in expanded expression of *sox2*, *sox11*, and *irx1*, whereas increased expression of Zmym4 (B2) caused no change in their expression in most embryos. Bar graphs are as in **(A)**. **(C)** In the neural crest, knockdown of Zmym4 (C1) resulted primarily in reduced expression of *foxd3* and *sox9*. Increased expression of Zmym4 (C2) primarily increased *foxd3* expression, and caused broader *sox9* expression in some embryos (∼35%; C2) and reduced expression in others (∼41%; D2). Bar graphs are as in **(A)**. **(D)** In the preplacodal ectoderm, knockdown of Zmym4 (D1) primarily reduced the expression of *six1, irx1, sox9*; in contrast, nearly equal numbers of embryos showed no change, expanded or reduced expression of *sox11*. Increased expression of Zmym4 (D2) showed a minor decrease in *six1*, whereas for *sox11*, *irx1* and *sox9* some embryos showed broader expression while others showed reduced expression. In the D2 embryo images, PPE expression of *six1* on mRNA-injected side is slightly reduced compared to control, *sox11* is reduced and *irx1* is reduced in the anterior placode (arrows). In D2, *sox9* is reduced in the otic placode on the injected side (red bar) compared to control (black bar); in C2, *sox9* is larger in the otic placode on the injected side (red bar) compared to control (black bar). Bar graphs are as in **(A)**. All embryo images are anterior views with dorsal to the top. np, neural plate. Black scale bars in top right corners = 300 μm.

At early neural plate stages (stages 13–14), in the majority of embryos knockdown (KD) of Zmym4 decreased the expression domains of *msx1* and *tfap2α* but expanded the *pax3* domain (Figure 3A, A1). At neural fold stages (stages 16–18), Zmym4 KD primarily expanded three NP genes (*sox2, sox11, irx1*; Figure 3B, B1) and reduced two NC genes (*foxd3*, *sox9*; Figure 3C, C1). Zmym4 KD reduced the domains of three PPE genes - *six1, sox9*, *irx1*—in the majority of embryos. In contrast, the *sox11* PP E domain was reduced in about a third of embryos, expanded in a third and unchanged in a third (Figure 3D, D1). Thus, loss of Zmym4 has a significant impact on the expression domains of genes required for the proper patterning of the embryonic ectoderm that gives rise to the central and peripheral nervous systems and several craniofacial sensory structures.

To determine whether increasing Zmym4 protein above endogenous levels altered early gene expression, *zmym4* mRNA was microinjected as described for the MOs. At early neural plate stages, only a minority of embryos showed either an increase or decrease in NB gene expression (Figure 3A, A2). Similarly, the majority of embryos showed no effect on the NP genes (Figure 3B, B2). In contrast, increasing levels of Zmym4 primarily expanded the NC domain of *foxd3* and about equally expanded or decreased the NC domain of *sox9* (Figure 3C, C2). Increased levels of Zmym4, however, had variable effects on PPE genes. The *six1* domain showed a minor decrease in most embryos (Figure 3D, D2). Although many embryos showed decreased levels of *irx1* (36%) or *sox9* (50%) PPE expression, other embryos showed expanded domains (Figure 3D, D2). Similar to the effects of its KD, increased Zmym4 protein resulted in about equal proportions of embryos with decreased, increased or no change in the *sox11* PPE expression domain (Figure 3D, D2).

Together, these results suggest that loss of Zmym4 alters the expression of NB genes that results in expansion of the NP genes at the expense of NC and PPE genes. These data are consistent with Zmym4 being important for early brain development (Xiang et al., 2020), but also suggest a required function in the development of the NC and PPE. Increased expression of Zmym4, on the other hand, had only small effects on either the NB or NP, but instead expanded NC genes seemingly at the expense of PPE genes. One explanation could be that Zmym4 levels after 100 pg mRNA injections were not increased sufficiently for an effect. To test this, we doubled the mRNA concentration (200 pg) but detected no statistically significant differences from the 100 pg injections (*p* > 0.05; Chi-square test; data not shown). Therefore, we propose that Zmym4 targets may be sensitive to threshold amounts of protein and exceeding those levels may not affect the frequencies at which we observe the assessed phenotypes.

### 3.3 Loss of Zmym4 results in dysmorphology of the craniofacial cartilages

**FIGURE 4:**
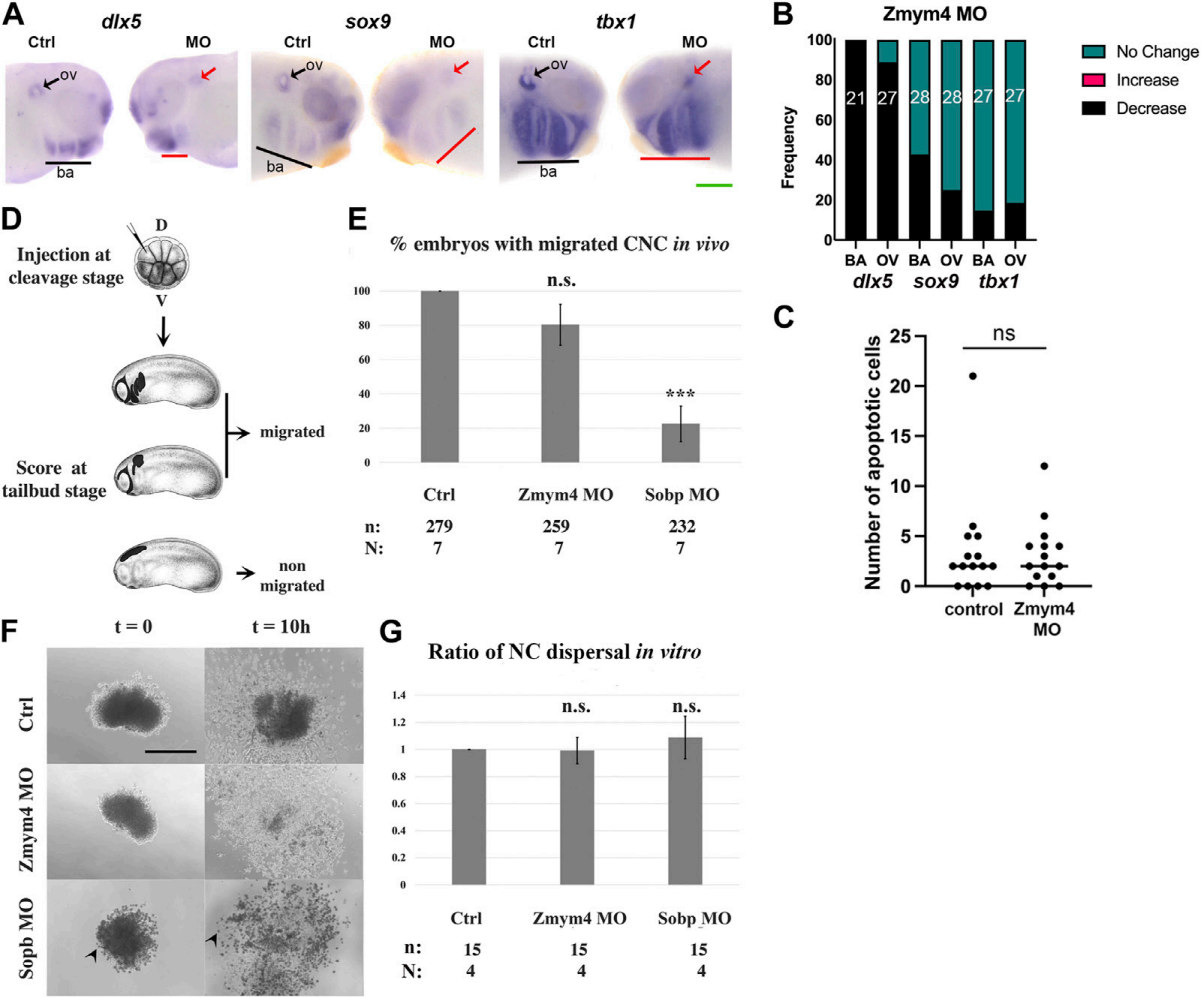
Loss of Zmym4 caused reduced otic vesicle and branchial arch gene expression. **(A)** Knockdown of Zmym4 reduced the expression *dlx5*, *sox9*, and *tbx1* in larval otic vesicles (ov, arrows) and branchial arches (ba, underlined) on the MO-injected side compared to the control (Ctrl) side of the same embryo. Green scale bar in lower right corner indicates 200 μm. **(B)** The percentages of embryos that showed “no change” (green), “increased” (pink), or “decreased” (black) expression on the MO-injected side compared to the control side of the same embryo; the number of embryos examined for each gene is indicated within the bar. Zmym4 knockdown reduced the OV and BA expression of *dlx5* in nearly every embryo, whereas it reduced *sox9* or *tbx1* expression only in small percentages of embryos. **(C)** Scatter plot of the number of apoptotic cells counted on the control and MO-injected sides of Zmym4 morphant larvae (n = 15). There was no significant difference (ns = *p* > 0.05) in the number of apoptotic cells after loss of Zmym4. **(D)** Blastomeres on the dorsal **(D)** animal side of cleavage stage embryos were microinjected with *mbGFP* mRNA ± Zmym4 MOs or Sobp MOs. At tailbud stages, embryos were scored as “migrated” if some fluorescently labeled NC entered a migratory pathway and as “non-migrated” if none of them entered any pathway. Embryo images downloaded from Xenbase (https://www.xenbase.org/xenbase/zahn.do). **(E)** The percentage of embryos with migrated NC cells in the assay shown in **(D)** n = the number of embryos analyzed; N = the number of independent trials. n. s. = not significant compared to controls. ***, *p* < 0.001, Student’s t-test **(F)** DIC images of typical cranial NC explants when they first adhered to the substrate (t = 0) and 10 h later (t = 10h). Arrowheads indicate Sobp morphant cells that are rounded rather than flattened on the FN substrate. Scale bar = 200 μm. **(G)** The ratio of the area the NC covered between the start and end of the culture period (end/beginning) (NC dispersal). n. s. = not significant compared to controls (*p* > 0.05, Student’s t-test). n = the number of embryos analyzed; N = the number of independent trials.

Since loss and gain of Zmym4 had opposite effects on NC gene expression and mostly reduced that of PPE genes, we asked whether those changes resulted in later disruptions of gene expression in the structures derived from these progenitor fields: the otic vesicle (OV), which is of placode origin, and the BAs, whose mesenchymal core is derived from cranial NC. Both the OV and BA express genes that are required for NC migration and chondrogenesis, including *dlx5, sox9,* and *tbx1* (Acampora et al., 1999; Depew et al., 1999; Garg et al., 2001; Spokony et al., 2002; Mori-Akiyama et al., 2003; Saint-Germain et al., 2004; Brown et al., 2005; Moraes et al., 2005; Tazumi et al., 2010). Zmym4 KD decreased the intensity and domain size of *dlx5* in both the OV and BAs (Figures 4A,B). In about 75% of these embryos all BA were affected and in 25% only the posterior BAs were affected, as illustrated in Figure 4A. Loss of Zmym4 had less of an effect on *sox9* or *tbx1* expression. *sox9* was decreased in the OV in 25% of embryos and in the BA in 43% of embryos, whereas *tbx1* was decreased in the OV in 19% of embryos and in the BA in 15% of embryos (Figures 4A,B). Because many deficiencies in cranial NC after loss of gene function can be attributed to increased apoptosis (*e.g.*, Jones et al., 2008; Ross and Zarbalis, 2014), we performed a TUNEL analysis at late larval stages (∼stages 35–36), when NC migration into the BAs is complete. KD of Zmym4 caused no significant difference in the number of TUNEL positive cells between the control BAs (mean = 3.13 cells, N = 15) and the morphant BAs (mean = 3.53 cells; *p* > 0.05) (Figure 4C). These results indicate that the reduction of *dlx5*, *sox9* or *tbx1* BA expression domains were not due to cell death.

To determine whether the reduction in BA gene expression in Zmym4 morphants was due to delayed NC migration into the BAs, we performed *in vivo* and *in vitro* NC migration assays. For the *in vivo* assay, the presence or absence of GFP-labeled NC in the branchial arch migratory pathways was assessed at stage 25 in both Sobp and Zmym4 morphants, as described in Weir et al. (2021) (Figure 4D). We found that Sobp KD led to a significant decrease in the percentage of embryos that contained NC cells in any of the migratory streams (Figure 4E), as expected from the previously reported craniofacial defects (Tavares et al., 2021). In contrast, Zmym4 KD did not lead to a significant decrease compared to controls (Figure 4E). For the *in vitro* assay, explanted cranial NC derived from control, Zmym4 morphant or Sobp morphant embryos were assessed by time lapse microscopy. The area occupied by the cells of the explant at the beginning (Figure 4F t = 0) and at the end (Figure 4F t = 10) of the culture period was measured and the ratio (end/beginning) calculated and normalized to the ratio obtained from control embryo explants. In each of the three conditions, the initial explants and the cells derived from them attached to the FN substrate (Figure 4F). The only difference was that the control cells and the Zmym4 morphant cells flattened as they dispersed from the explant, whereas the Sobp morphant cells remained rounded throughout the time course of the assay (Figure 4F, arrowheads). Consistent with the *in vivo* assay, Zmym4 KD did not lead to a significant change in the area occupied by NC cells that migrated away from the explant (Figure 4G). Although Sobp KD cells did not flatten on the FN substrate, they also dispersed over an area that was not significantly different from controls (Figure 4G). Together, these NC migration assays show that Zmym4 KD does not appear to affect the ability of NCs to migrate into the BAs.

**FIGURE 5:**
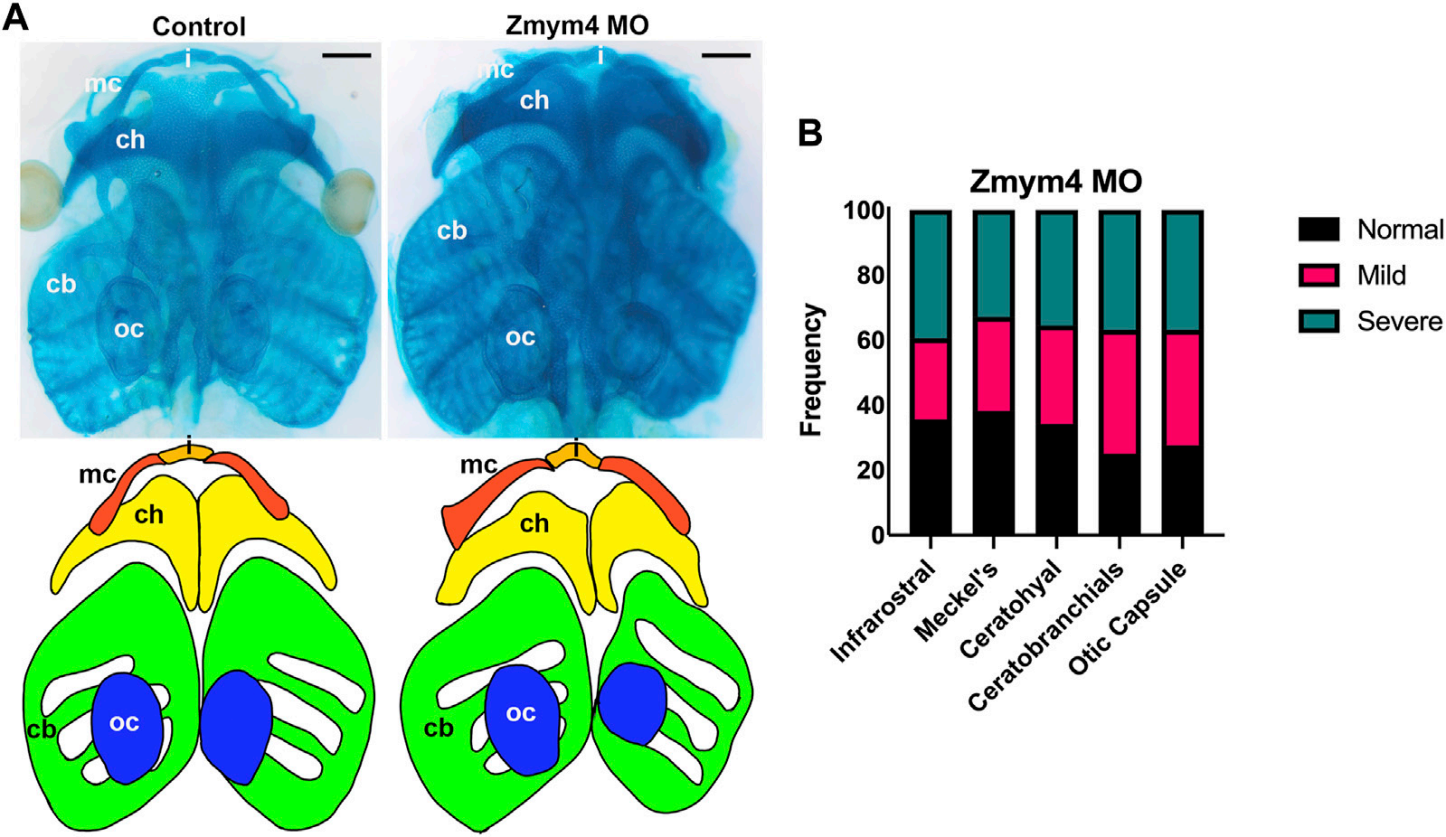
Loss of Zmym4 results in craniofacial cartilage dysmorphologies. **(A)** Top: ventral views of Alcian blue stained heads from a control tadpole and a sibling Zmym4 morphant (Zmym4 MO). Loss of Zmym4 on the right side of the tadpole resulted in compression of the infrarostral cartilage **(I)**, thickening and shortening of the Meckel’s cartilage (mc), shortening and compression of the ceratohyal cartilage (ch) and reduced size of the ceratobranchial cartilages (cb). The otic capsule (oc) also was reduced in size on the injected side. Bottom: tracing of the craniofacial cartilages to better demonstrate the differences in morphology between the control and morphant infrarostral (orange), Meckel’s (red), ceratohyal (yellow), ceratobranchial (green) and otic capsule (blue) cartilages. Black scale bars in top right corners = 200 μm. **(B)** Bar graph depicting the percentage of Zmym4 morphants (n = 76) that displayed either “normal” (black), mildly affected (pink), or severely aberrant (green) morphology for each of the five craniofacial cartilages analyzed.

Since *dlx5, sox9,* and *tbx1* also are required for cranial chondrogenesis (Depew et al., 1999; Garg et al., 2001; Mori-Akiyama et al., 2003; Tazumi et al., 2010), we assessed whether their altered BA expression at larval stages would lead to altered morphology of the craniofacial cartilages. To test this, Zmym4 morphants were grown to tadpole stages and their cartilages stained with Alcian blue. Loss of Zmym4 resulted in dysmorphology of the craniofacial cartilages, marked by compression of the infrarostral cartilage, thickening and shortening of Meckel’s cartilage, compression of the ceratohyal, and reduced size of the otic capsule and ceratobranchial cartilages (Figure 5A). When individual cartilages were measured and scored for severity of morphological defect, we found that the majority of tadpoles examined (n = 76) had at least a mild defect on the MO-injected side (Figure 5B). It is not surprising that many craniofacial cartilages were affected by Zmym4 KD since reduced *dlx5* expression was observed in most of the branchial arches at tailbud stages (75%); perhaps those tadpoles in which the cartilages appeared normal (∼25–30%; Figure 5B) represent the larvae (∼25%) in which reduced *dlx5* was only observed in the posterior BAs, as shown in Figure 4A. Taken together, these data confirm that Zmym4 is required for normal development of the NC-derived craniofacial cartilages.

## 4 Discussion

Most of the cranial sensory and skeletal tissues are derived from neural tube, neural crest and preplacodal ectodermal stem cells during embryogenesis. It is hoped that by understanding the molecular and cellular interactions that result in the formation of these different stem populations, regenerative therapies may be developed to treat the large number of known syndromic and non-syndromic craniofacial dysmorphologies. Our long-standing interest in this quest has been to reveal the molecular interactions between the Six1 transcription factor and putative interacting partners that may account for variable craniofacial malformations found in Branchio-oto-renal syndrome (BOR) patients that include: the outer, middle, and inner ear; branchial fistulas and cysts; and in some cases renal abnormalities (Moody et al., 2015; Smith, 2018). Approximately 5% of patients presenting with BOR have a mutation in *SIX1*, and approximately 45% present with a mutation in *EYA1*, which encodes a SIX1 co-factor that is required for transcriptional activation (Buller et al., 2001; Ruf et al., 2004; Lee et al., 2007; Sanggaard et al., 2007; Patrick et al., 2009; Li et al., 2010; Moody and Saint-Jeannet, 2014; Moody and LaMantia, 2015; Klingbeil et al., 2017; Smith, 2018). We hypothesized that since nearly half of patients have a mutation in *EYA1*, it is likely that unidentified SIX1 interacting proteins may be causative (Moody et al., 2015). To this end, we previously screened the fly interactome for binding partners of the *Drosophila* homologue of Six1 whose orthologs are also expressed in *Xenopus* embryos (Neilson et al., 2010). These screens identified new Six1 cofactors, including Proliferation-associated 2G4 (Pa2G4), Microspherule protein 1 (Mcrs1), and Sine oculis binding protein (Sobp) (Neilson et al., 2017; Neilson et al., 2020; Tavares et al., 2021; Keer et al., 2022). This screen also identified zinc finger MYM-containing 4 (Zmym4) as a potential Six1 cofactor based on its structural similarities to Box2 and Box3 of Sobp (Kenyon et al., 2005; Neilson et al., 2010). In this report, we tested whether Zmym4 can interact with Six1, affect its transcriptional activity and/or is required for the development of the embryonic stem populations of important craniofacial structures.

### 4.1 Zmym4 does not interact directly with Six1 but may act as a pioneer or scaffold protein

We found no evidence in the Co-IP or luciferase assays that we used that Zmym4 binds to Six1 or alters its transcriptional activity, thus indicating it is unlikely to be a *bona fide* Six1 cofactor. Nonetheless, the KD experiments demonstrate that Zmym4 is required for the proper apportioning of gene expression domains that divide the embryonic ectoderm into neural plate, neural crest and cranial placodes. While there is no direct evidence yet for the manner by which Zmym4 regulates cranial gene expression, work on two highly similar proteins, ZMYM2 and ZMYM3, allow us to speculate that Zmym4 may have a developmental function in transcriptional control by remodeling chromatin.

ZMYM2 interacts with the fibronectin type-III (FNIII) domain of ATF7IP, which is required for the efficient transcriptional silencing of both genes and transposable elements by the SETDBI complex (Tsusaka et al., 2020). The FNIII domain is similar to an “ITEFSL” sequence found within MYC homology box II that enables histone acetylation and is essential for FNIII domain interactions (Kalkat et al., 2018; Tsusaka et al., 2020). ZMYM4 also possesses an ITEFSL-like sequence (Supplementary Figure S3), suggesting that it also may interact with the FNIII domain of ATF7IP (Tsusaka et al., 2020). ZMYM3 serves as a scaffolding protein that coordinates interactions between deacetylases, demethylases, and RNase H type enzymes (including HDAC and CoREST), and the eighth and ninth zinc fingers of ZMYM2 protein can bind directly to the LSD1-CoREST-HDAC complex (Shapson-Coe et al., 2019). As RNase H2 can interact with both ZMYM2 and ZMYM4, it has been suggested that ZMYM4 may also interact with the LSD1-CoREST-HDAC complex to mediate transcriptional regulation (Jabbari et al., 2018; Shapson-Coe et al., 2019). Furthermore, ZMYM2 and ZMYM3 contain a new class of SUMO-interacting motifs (SIMs) that can interact with SUMO2 in a 1:1 stoichiometry, and recent studies show that ZMYM4 is also SUMOylated (Anderson, 2015; Lamoliatte et al., 2017; Cibis et al., 2020). Both ZMYM2 and ZMYM4 are B-MYB binding proteins, although the interaction between ZMYM4 and B-MYB does not appear to be direct (Cibis et al., 2020). Nonetheless, a recent MYB chromatin immunoprecipitation mass spectrophotometry (ChIP-MS) assay identified that only MYB and seven other proteins were specifically enriched in this assay, one of which was ZMYM4 (Yong et al., 2023). Thus, given the similarities with ZMYM2 and ZMYM3, we predict that Zmym4 may accomplish its effects on cranial gene expression by modulating or stabilizing interactions between complexes that modify chromatin availability, perhaps allowing it to act as a pioneer factor during early development.

We found that loss of Zmym4 at neural border stages decreased the expression of NB genes (*msx1*, *tfap2α*) and neural plate genes (*sox2*, *sox11*, *irx1*)*,* whereas its overexpression had minimal effects on them. These results are consistent with Zmym4 acting as a pioneer factor in the early neural ectoderm. Pioneer transcription factors implement new cell fates during development by binding to DNA target sites in a closed chromatin configuration and modulating active-chromatin histone modifications that remodel the chromatin into an open configuration, thus making critical sites accessible to other transcription factors (Balsalobre and Drouin, 2022). As such, loss of a pioneer factor typically causes significant changes in downstream gene expression, but overexpression of the same factor typically results in little to no change in later gene expression because the endogenous protein has already opened the chromatin, making critical binding sites already available. An important next step will be to biochemically determine whether Zmym4 acts as a chromatin remodeling/pioneer protein that regulates early cranial gene expression.

### 4.2 Zmym4 may act as a transcription factor in NC and PPE stem populations

Interestingly, the effects of loss- and gain-of-function of Zmym4 on NC and PPE genes were quite different from those on NB and NP genes. Loss of Zmym4 resulted in the reduction *foxd3* and *sox9* expression in the premigratory NC stem population, whereas its overexpression resulted primarily in broadening their expression domains. These data would suggest that Zmym4 may act as a NC-promoting transcription factor, which is consistent with our identification of a DUF3504 domain in the C-terminal region of Zmym4 (Figure 1; Supplementary Figure S3). DUF3504 domains recently were shown to exhibit *in silico* structural similarities and evolutionary conservation with BEN family DNA-binding domains, suggesting they have the ability to serve as a transcription factor (Pan et al., 2023). ZMYM4 also has downstream alternative initiation sites (dATI), which are characterized by having an AUG codon downstream of the canonical initiator methionine codon that can be used to generate multiple proteins from the same mRNA sequence and to traffic proteins to different cellular compartments (Kazak et al., 2013). In ZMYM4, these dATI sites have been predicted to function in transcriptional regulation, supporting the possibility that Zmym4 could function in this way in the NC stem population (Kazak et al., 2013).

The potential function of Zmym4 as a PPE-determining transcription factor is less clear. Both loss- and gain-of-function resulted primarily in a reduction in the expression of *six1*, whose protein is a key regulator of the other PPE genes. Zmym4 KD resulted primarily in reduced *irx1* and *sox9* domains; overexpression of Zmym4 caused reduction to a lesser extent and broader domains in a smaller percentage of embryos. Both loss- and gain-of-function resulted in nearly equal proportions of reduced and broadened expression of *sox11*. Since the formation of the PPE is regulated by a complex gene regulatory network that differentially effects each participating gene (Klein et al., 2002; Grocott et al., 2012; Moody and LaMantia, 2015; Riddiford and Schlosser, 2016; Hintze et al., 2017; Maharana and Schlosser, 2018), an important next goal will be to sort the direct and indirect effects of Zmym4 KD on each element in the network.

It is worth noting that ZMYM4 also has been shown to have numerous functions outside of direct transcriptional control, the details of which are only now being uncovered. These include: 1) ZMYM4-AS1, a long non-coding RNA that can be used as a competitive endogenous RNA to regulate gene expression levels and affect cell function by competing with microRNAs (Li et al., 2021), 2) a circular RNA form (*i.e.*, circ_0011536) that may function as a microRNA sponge in colorectal and endometrial cancers (Cheng et al., 2018; Yin et al., 2022), and 3) fusion with a rolling-circle transposon that generates an alternative transcript of ZMYM4 that contains a cryptic splice site, an initiation codon, and a few codons in the predicted ORF from the transposon donor (Thomas et al., 2014). Therefore, the possible functions of Zmym4 during early cell fate decisions underlying the apportioning of the embryonic ectoderm into the various stem populations that contribute to craniofacial structures - the neural plate, neural crest, preplacodal ectoderm, and the non-neural ectoderm—remain to be determined by future in-depth studies.

### 4.3 Early loss of Zmym4 causes defects in craniofacial chondrogenesis

Given our finding that loss of Zmym4 results in reduction of NC and PPE gene expression, we investigated whether KD of Zmym4 had effects on later derivatives of these stem populations, the OV - derived from the otic placode - and the BA- derived from the cranial neural crest. Consistent with the gene expression data at neural fold stages, we found that loss of Zmym4 resulted in reduced expression of *dlx5*, *sox9* and *tbx1* in the OV and BA at larval stages. At tadpole stages, these gene expression changes persisted as dysmorphologies in the craniofacial cartilages. One potential cause of the reduction in gene expression domains and cartilage element is errors in migration of the NC into the BA, especially since Sox9 and Tbx1 are required for this migration (Spokony et al., 2002; Moraes et al., 2005). However, we did not detect any defects using either an *in vivo* or *ex vivo* assay. Another potential cause is increased apoptosis during BA formation; again, we did not detect a significant difference in the number of apoptotic cells in the BA between controls and Zmym4 morphants. A third potential cause is reduced cell proliferation either prior to or during migration of the NCs. Although we did not test this directly, KD of ZMYM4 previously was shown to have no obvious effects on cell cycle progression (Cibis et al., 2020). Therefore, the possible functions of Zmym4 during craniofacial chondrogenesis remain to be determined.

### 4.4 Conclusion

Our study is the first to identify a role for Zmym4 during early cranial gene expression and later craniofacial chondrogenesis. While very little is currently known about the function of ZMYM4 itself, its closely related proteins ZMYM2 and ZMYM3 have been shown to contain non-canonical SIMs and are important in chromatin remodeling either directly (ZMYM2) or by stabilizing the interactions between the LSD1-CoREST-HDAC repressive complex and chromatin by acting as scaffolding (ZMYM3). As ZMYM4 has been identified as being critical for early brain development (Xiang et al., 2020) and our data show that it is required for the proper apportioning of the embryonic ectoderm into neural plate, neural crest and PPE, it will be important to determine if Zmym4 accomplishes this by regulating chromatin accessibility during neural development, especially by facilitating the cell fate change from a multipotent state to a more restricted one. Data from human cell lines also indicate that ZMYM4 may have a direct transcriptional function (Kojima and Jurka, 2011; Pan et al., 2023), which we predict may impact the expression of neural crest and placode genes. Although we have yet to uncover the molecular mechanism(s) by which this multifunctional protein regulates these processes, taken together, the data presented herein demonstrate that Zmym4 plays an important role in regulating craniofacial development. They also identify exciting avenues of future study into the potential roles of this factor in the pathophysiology of craniofacial dysmorphologies.

## Data Availability

The original contributions presented in the study are included in the article/Supplementary Material, further inquiries can be directed to the corresponding author.
