# Supplementary material for: Zmym4 is required for early cranial gene expression and craniofacial cartilage formation
Source: Front Cell Dev Biol. 2023 Oct 3;11:1274788. doi: 10.3389/fcell.2023.1274788 (PMC10579616; doi:10.3389/fcell.2023.1274788)
Supplement: Supplementary file 1 [file Table1.DOCX]

**Supplemental Figure legends**

**Supplemental Figure 1**: **Construction of Zmym4 plasmids and validation of MOs. A**) The gBlock fragment sequences that were used to generate the *pCS2+zmym4.L-5’UTR*, *pCS2+zmym4.L-5’UTR3’HA*, *pCS2+zmym4.L-5’MOins*, *pCS2+zmym4.L-5’MOins3’HA*, and *pCS2+zmym4.L-3’HA* plasmid constructs are indicated. Sites of restriction enzymes, morpholino binding and HA tag are color coded. **B**) Western blot validation that HA tagged protein encoded by *pCS2+zmym4.L-3’HA*, *pCS2+zmym4.L-5’UTR3’HA*, and *pCS2+zmym4.L-5’MOins3’HA* plasmids is efficiently expressed. Arrow indicates appropriately sized band. **C**) Western blot image of 5μg of lysates from uninjected control and MO-injected sibling embryos stained with rabbit anti-Zmym4 (green) and mouse anti-αTubulin (red) demonstrating a reduced level of Zmym4 protein in Zmym4 morphant embryos. **D**) Efficiency of Zmym4 protein knockdown in embryos by MOs averaged from two replicate experiments. **E**) *In situ* hybridization for *sox9* after Zmym4 MO injection on the embryo’s left side (MO; right side of image). MO injection alone result in reduced *sox9* expression in the neural crest of ~96% of embryos. This loss of *sox9* expression was significantly reduced (~15%) in most embryos (Rescue) that were co-injected with mRNA encoding the MO-insensitive Zmym4 construct (*zmym4.L-5’MOins3’HA)*.

**Supplemental Figure 2**: Image of an Alcian blue stained control tadpole demonstrating where measurements were collected for the size of the infrarostral (i), Meckel’s (mc), ceratohyal (ch), and otic capsule (oc) cartilages.

**Supplemental Figure 3: Dotplot showing the sequence similarity of Zmym4 between the long and short homeologs in *Xenopus laevis*, *Xenopus tropicalis*, the human protein, and Without children (Woc), the *Drosophila melanogaster* ortholog.** The nine zinc finger (ZF) domains identified in the human protein are highlighted, each in a different color. All of the ZF domains are highly conserved between species with the exception of the first ZF domain, which is considerably longer in *Drosophila.* The areas of homology with Sine oculis binding protein (Sobp) Box2 and Box3, which were used to identify Zmym4 as a potential Six1 binding partner are outlined by boxes. The ITESFL-like domain is indicated in pink and the DUF3504 domain in yellow. The sequence alignments were generated using Tcoffee (Notredame *et al*., 2000) and assembled into Dotplots using ESPript (Robert & Gouet, 2014).

**Supplemental Figure 4: Zmym4 does not alter the transcriptional activity of Six1 BOR variants either alone or in the presence of Eya1.** Luciferase activity of the pGL3-6XMEF-luciferase reporter in HEK293T cells transfected with different combinations of plasmids expressing empty vector control, wild type Six1, Eya1, Zmym4, or BOR variants of Six1 (V17E, R110W, Y129C). Data are normalized to Renilla expression with a constitutive promoter. **A**) The level of luciferase activity was not significantly different between wild type Six1 or any of the BOR1 variants in the presence of Zmym4. Transcriptional activation by wild type Six1+Eya1 is a positive control for the assay. **B**) While luciferase activity is significantly increase over control in the presence of wild type Six1+Eya1, this level was not significantly changed by the addition of Zmym4. Further, none of the BOR variants of Six1 significantly activate transcription in the presence of Eya1 + Zmym4.
