# Supplementary figures and images for: Zmym4 is required for early cranial gene expression and craniofacial cartilage formation
Source: Front Cell Dev Biol. 2023 Oct 3;11:1274788. doi: 10.3389/fcell.2023.1274788 (PMC10579616; doi:10.3389/fcell.2023.1274788)

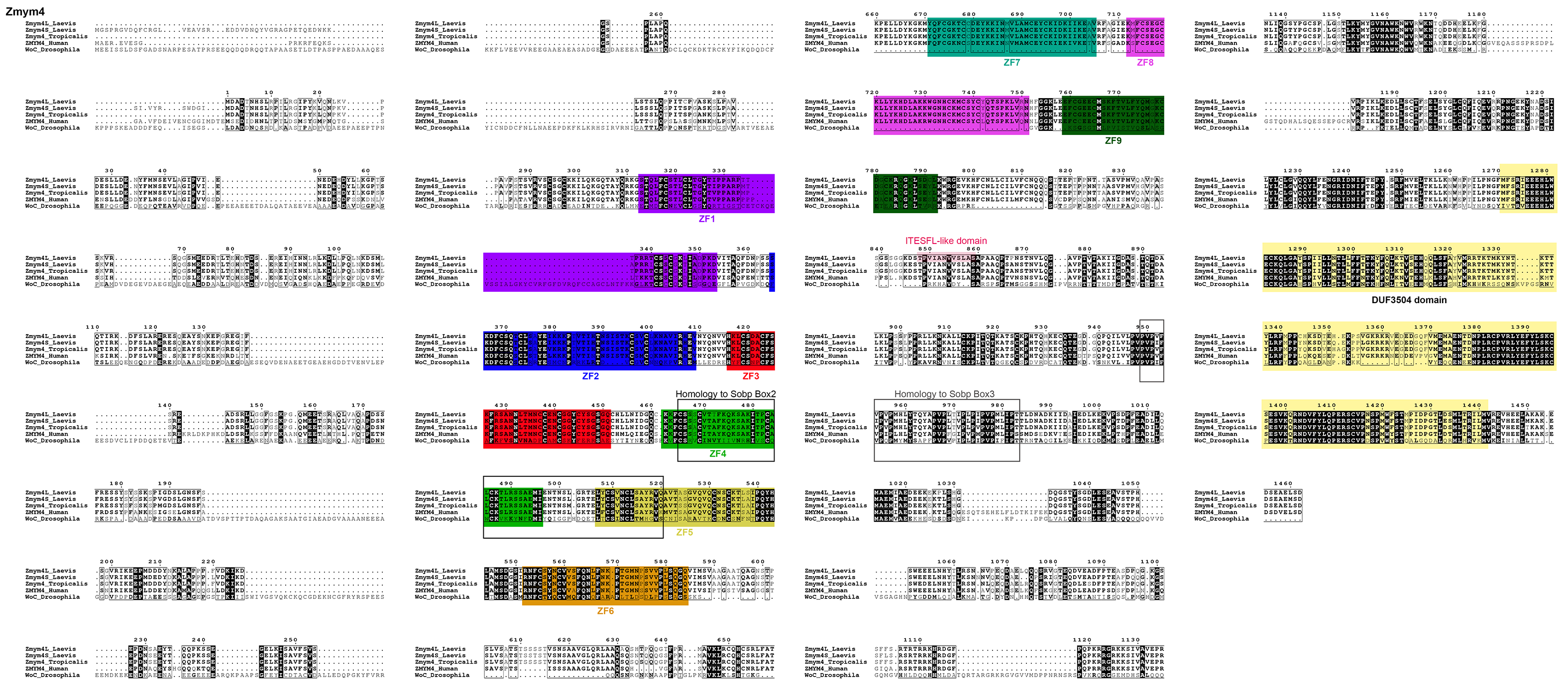

Supplement: Supplementary file 2 [file Image3.TIF]

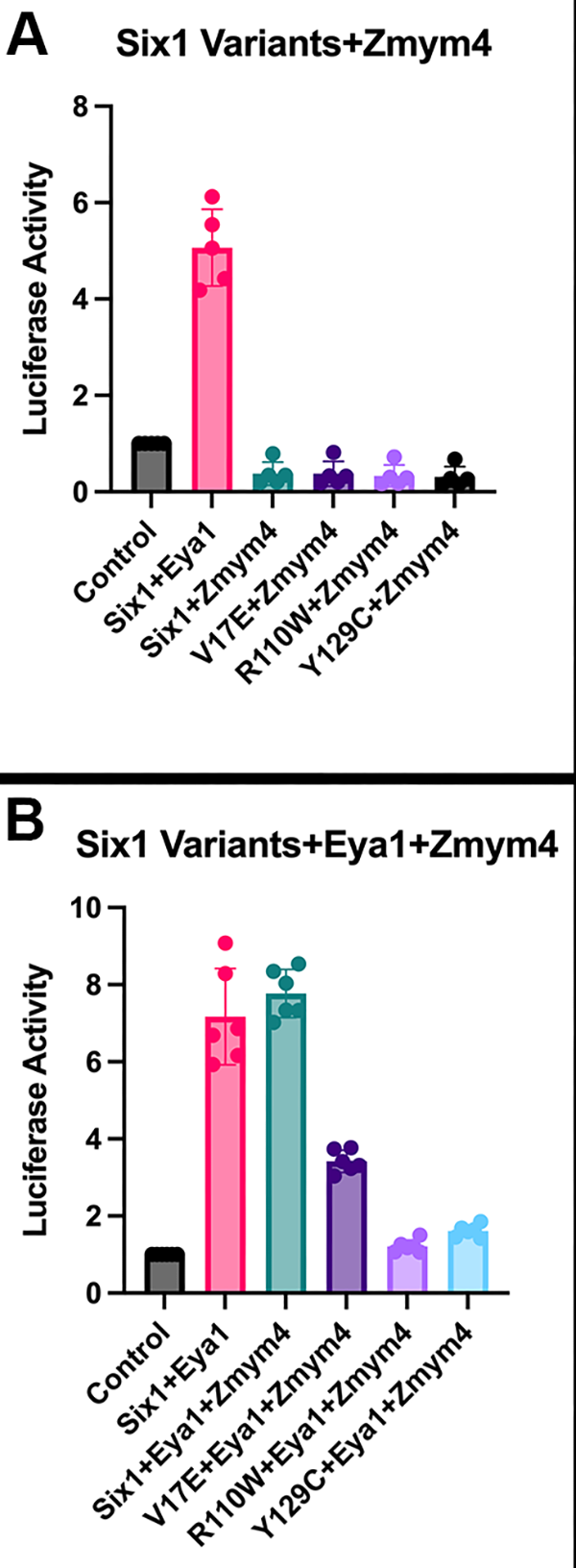

Supplement: Supplementary file 3 [file Image4.TIF]

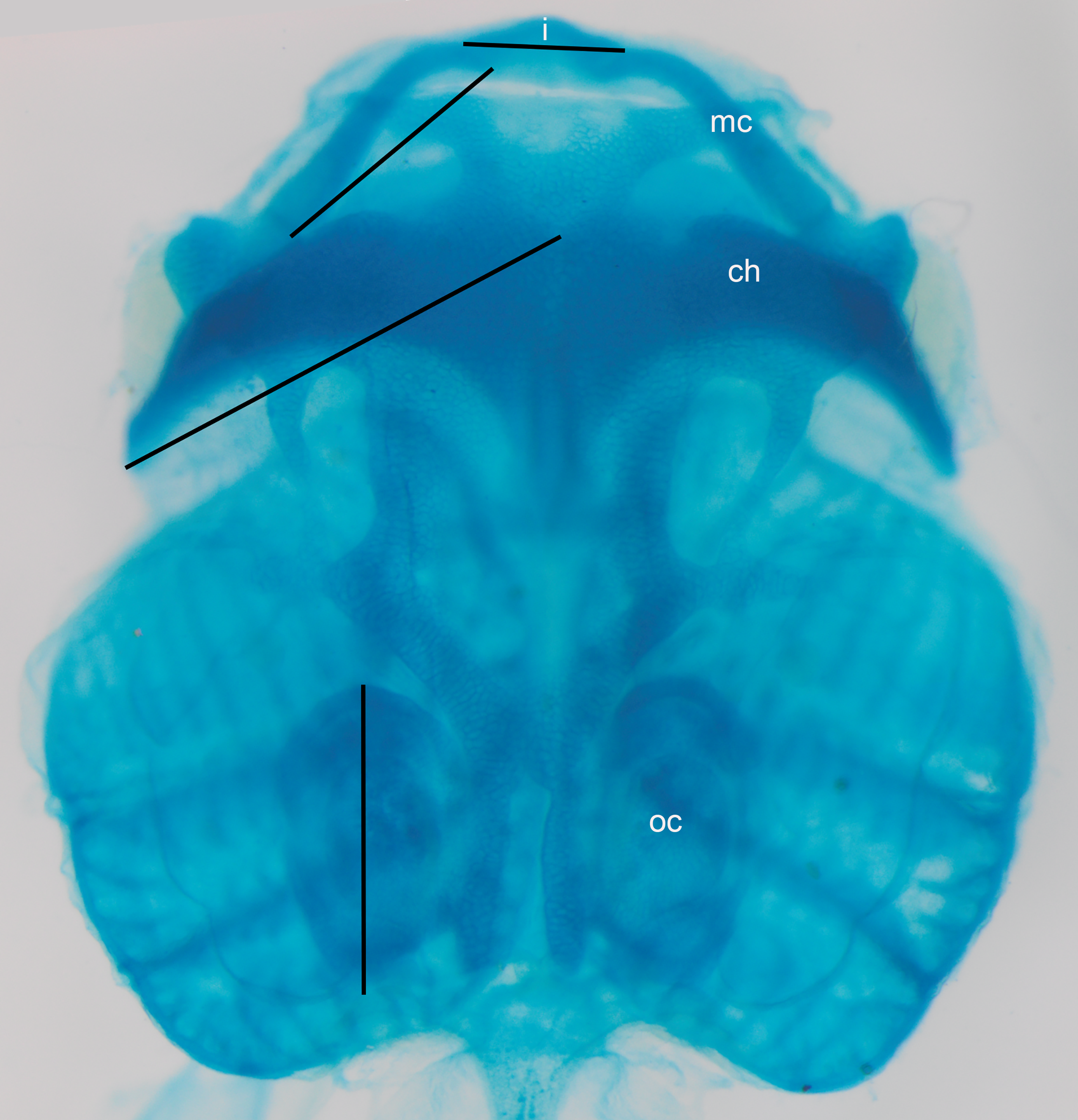

Supplement: Supplementary file 4 [file Image2.TIF]

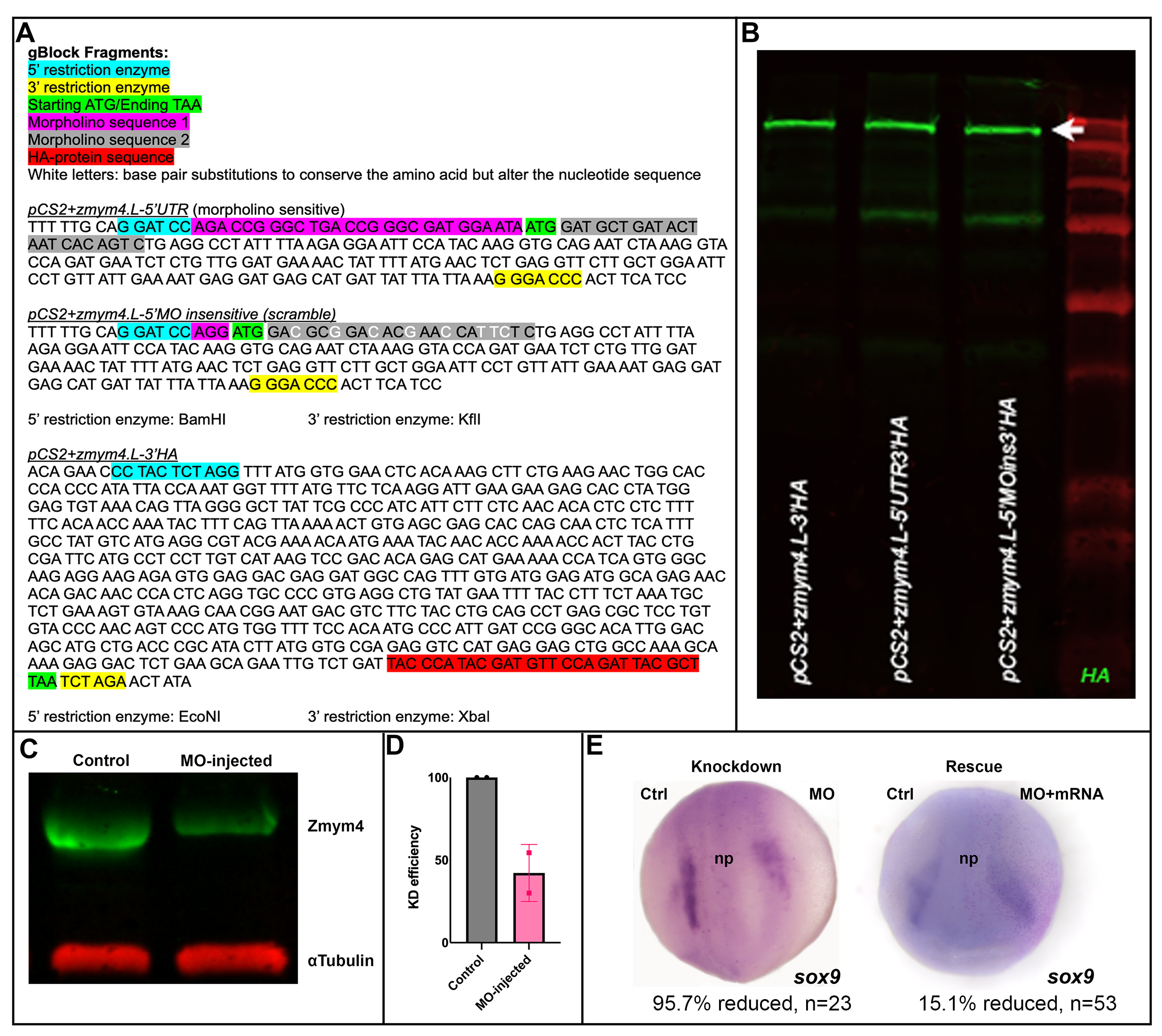

Supplement: Supplementary file 5 [file Image1.TIF]
